# Strengthening research capacity in LMICs to address the global NCD burden

**DOI:** 10.1080/16549716.2020.1846904

**Published:** 2020-12-29

**Authors:** Arianne Malekzadeh, Kathleen Michels, Celia Wolfman, Nalini Anand, Rachel Sturke

**Affiliations:** Fogarty International Center, National Institutes of Health, Bethesda, MD, USA

**Keywords:** Noncommunicable diseases, capacity building, global health, scientific research, research training

## Abstract

The burden of noncommunicable diseases (NCDs) continues to rise across the globe, and the risk of dying prematurely from an NCD in a low- and middle-income country (LMIC) is almost double that in a high-income country. Confronting this crisis requires a critical mass of scientists who are well versed in regional health problems and understand the cultural, social, economic, and political contexts that influence the effectiveness of interventions to address NCDs. Investing in research capacity strengthening in LMICs is critical to effectively combating disease, and local researchers are best poised to address the health challenges in their home countries given their understanding of the unique culture and context in which they are working. The Fogarty International Center of the U.S. National Institutes of Health has a set of programs focused on building individual and institutional NCD research capacity in LMICs. The Programs provide models for sustainable scientific research capacity strengthening, innovative funding mechanisms and partnership-building approaches. Investing in the training and scientific capacity of LMIC individuals and institutions not only helps foster a research culture and solidify local ownership of research, but it also ensures that the most appropriate solutions are developed, increasing the likelihood that those solutions will sustain over time. In addition, the Programs’ investigators have advanced the science across a range of NCDs and associated risk factors. This article describes key lessons and compelling cases from the Programs that can be harnessed by other health researchers and funders to further the global response to the NCD burden.

## Background

Noncommunicable diseases (NCDs) kill 41 million people each year. Seventy-one percent of all deaths globally are due to chronic illnesses including cardiovascular diseases, cancers, chronic respiratory diseases, and diabetes [1]. Over three quarters of these deaths occur in low- and middle-income countries (LMICs) and the risk of dying prematurely from an NCD in a LMIC is almost double that in a high-income country (HIC) [2]. The global burden of chronic diseases is expected to increase due to, among other factors, an aging population, rapid urbanization, the pollution of air, soil and water [3] and unhealthy lifestyles. If we are to reduce by one-third premature mortality from NCDs by 2030, a mark set by the United Nations Sustainable Development target 3.4 [4], swift action must be taken by stakeholders across the globe. However, many countries lack data and the human and research capacity to do what is necessary to tackle the growing NCD burden. In such places, research training, implementation research and research capacity building initiatives are badly needed [2].

The Fogarty International Center (FIC) of the National Institutes of Health (NIH) supports and facilitates global health research, builds international partnerships among research institutions, and trains the next generation of scientists to address major global health issues like the rise of NCDs. Through three research training programs – the International Clinical, Operational, and Health Services Research and Training Award Program (ICOHRTA), the Millennium Promise Awards: Noncommunicable Chronic Diseases Research Training Program (NCoD), and the Chronic, Noncommunicable Diseases and Disorders Research Training Program (NCD-Lifespan) – (henceforth called the ‘Programs’), FIC has helped build NCD research capacity specifically in LMICs by providing short- and long-term training opportunities for over 690 investigators and by funding institutional research capacity building. The Programs have not only advanced the science across a range of NCDs and associated risk factors, they also provide innovative funding models for effective partnership and research capacity building approaches [5]. This article describes key lessons and compelling cases from the Programs that can be harnessed to further the global response to the NCD burden.

## Noncommunicable diseases in low- and middle-income countries

In the last few decades, life expectancy in many LMICs has increased, largely due to declines in child mortality and public health measures that more effectively address and prevent infectious diseases [6]. These gains in life expectancy have been a key determinant of the growing prevalence of NCDs, lengthening the amount of time adults are exposed to or will live with NCD risk factors such as environmental threats, tobacco use and unhealthy diets [5]. Shifts in urbanization and globalization have further helped spur the rise of NCDs in LMICs as more people are exposed to indoor and outdoor pollution in densely packed cities and to toxic chemicals like pesticides, and have easier access to processed food and beverages, alcohol, and tobacco products [6]. NCDs are also increasingly occurring in younger populations in LMICs and resulting in worse health outcomes than is seen in HICs [7]. The resulting chronic diseases have high economic costs, due to the loss of productivity and steep health care expenses. In the absence of targeted solutions, it is estimated that NCDs could cost the global economy 47 USD trillion between 2011 and 2030 [8]. It is thus essential that health stakeholders take swift action to allay this universal problem.

## Research capacity strengthening

Solely funding scientific innovation, however, is not enough to make a lasting impact in this increasingly complex area of research. Investing in the research capacity of investigators and institutions is a critical component of combatting disease in LMICs. Local researchers are the best positioned to address the local health challenges of their own communities because they understand the unique culture and context in which they are working. Investing in the training and the scientific capacity of their institutions not only helps foster a research culture and solidify local ownership of the research, but it also ensures that the most appropriate solutions are developed, increasing the likelihood that those solutions are sustainable over time [9]. In a narrative review, Bowsher and colleagues find key factors that contribute to strengthened research capacity in LMICs: ‘addressing the individual, organizational and institutional level in tandem; adequate and sustainable funding and resources; capable and shared leadership within sustained and equitable partnerships; mentorship; the development of professional networks; and the linking of research to policy and practice, among others’ [10]. Research capacity can be strengthened through targeted investment, thoughtful mentorship programs, strong collaborations, and the application of monitoring and evaluation frameworks. Capacity building efforts should also include long-term planning and collaboration among a wide community of stakeholders [9], including researchers, program implementers, and policymakers at the local, state, and national levels. In a systematic review of health research capacity development approaches in LMICs [11], the authors posit that steady progress has been made in this area, including stakeholders being more reflexive of their actions and local stakeholders having more voice in and ownership of the research process. However, their findings suggest there is much room for improvement as few programs are dedicated to implementing systems- or institutional-level approaches to developing capacity. Many initiatives focus solely on high profile diseases; projects often lack operational and implementation research and quality evaluation data; and capacity development is often not prioritized due to an assumption that capacity will naturally develop when the research is conducted.

## Fogarty International Center programs

In response to the growing NCD burden and gaps in building sustainable NCD research capacity at LMIC institutions, FIC established three Programs over the course of the last two decades that support research training for U.S. and foreign scientists and the strengthening of global health research and international research collaborations. Between funding years 2001 and 2017, the Programs funded 79 awards at a total value of 79.4 USD million. The ICOHRTA Program was launched in 2001 in response to a gap in support for international training in clinical and health services research across a spectrum of infectious and noncommunicable diseases and disorders. In the mid-2000s, in alignment with the updated Global Burden of Disease study, the NCoD Program was created to address the growing burden of four NCDs – cancer, lung disease, diabetes, and cardiovascular disease – in LMICs in a more targeted way through specific research training opportunities. In 2011, the ICOHRTA and NCoD Programs were combined into a new NCD-Lifespan Program which continues today. The current program aims to 1) strengthen the research capacity of LMIC institutions so they can become national, regional and international centers of expertise in NCD research, 2) support multidisciplinary research training across the research continuum, 3) train a cadre of LMIC scientists in NCD-relevant research that will contribute to scientific advances and changes in clinical practice and public health policy, 4) support training-related research that is directly relevant to the health priorities of LMICs, 5) integrate with existing NCD research and public health programs in LMICs, and 6) strengthen core research support capabilities needed to manage grants at LMIC institutions. FIC’s partners from twelve NIH Institutes, Offices and Centers (ICOs) have been instrumental in the development and evolution of the Programs, contributing a total of 28.9 USD million to the Programs, 36% of the total costs [5]. Their collaboration reflects both the cross-cutting nature of the NCD challenge and the importance of creating diverse partnerships to address it.

## Programs’ outputs and impact

### Empirical evidence

**Figure 1.**
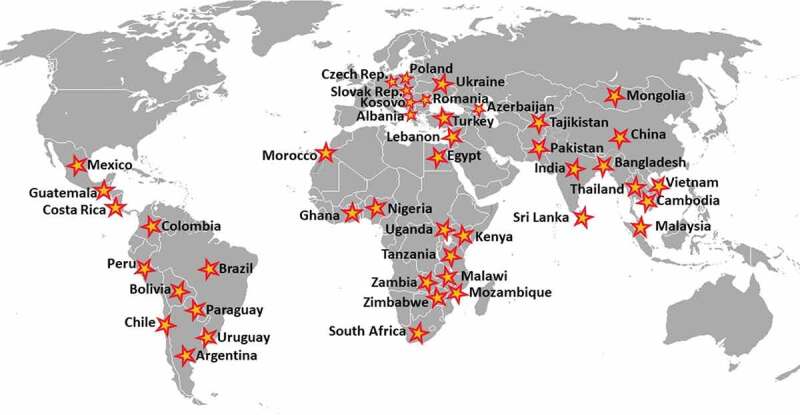
Awards by Country of Focus

**Figure 2.**
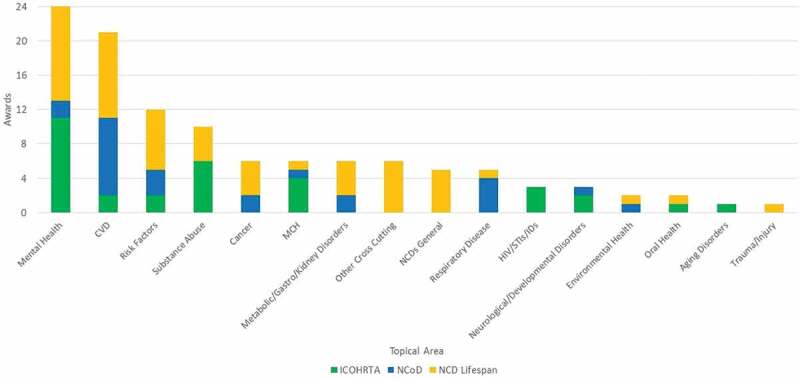
Research Topics of Funded Awards, 2001–2017.Grants can focus on more than one research area, and thus a grant can be counted in more than one NCD category

Notably, the Programs have contributed to the growing empirical evidence and research related to NCDs in LMICs. Specifically, the Programs supported research projects in 44 countries in 6 regions (see Figure 1) [5] on topics including mental health, cardiovascular and respiratory diseases, NCD risk factors, substance abuse, cancer, maternal and child health, environmental and oral health, trauma and injury, and aging, metabolic, gastro, kidney, neurological and developmental disorders (see Figure 2) [5].

From 2003 to 2017, a total of 982 publications citing a FIC NCD grant were published in scientific journals. The most common topics among the articles were mental health, NCD risk factors such as nutrition and obesity, and cardiovascular disease.

In 2018, FIC conducted a web-based survey of the Principle Investigators and primary foreign collaborators from 71 of the Program’s grants. (Eight grants were excluded from the survey, 5 of which were issued in 2017 and thus deemed too new to share outcomes, and 3 of which were a unique subset allowed to focus on HIV.) Seventy-three individuals responded to the survey, representing 51 of the 71 grants (72%), highlighting additional research outputs including those outlined in Table 1 [5].

**Table 1.** Examples of Research Outputs

Clinical Protocols
Forty-six percent of projects surveyed reported creating a clinical protocol to be used in an LMIC context, including:
– protocols for clinical trials of anti-cancer therapy in breast cancer patients
– provincial protocols for a) the assessment of first episode psychosis and prodromal status, and b) the assessment of untreated psychosis and reduction of untreated psychosis
– a WHO Mental Health Gap Action Program-based protocol for use by health providers
– a research protocol for the management of sickle cell disease
– protocols for air sampling and analyses in Mongolia
– adapted protocols for primary care physicians in Ukraine treating patients with addiction
– protocol for clinical use of dental prostheses for periodic uptake of fluoride
Patient Registries/Databases
Forty-two percent of projects surveyed implemented new patient registries or databases, collecting information ranging from stroke incidence in Peru to biospecimens in China to alcohol addiction in Poland. In Nigeria, grantees developed a Food Frequency Questionnaire that aggregates dietary intake over time and a validation tool through which they can assess the collected data.
Analytic Tools
A grantee group in Thailand made a software application to assist in the analysis of large national health data sets for diabetes studies. Another team designed a data analysis platform for genetic studies that can be used to identify genotype-phenotype relations in genome-wide association studies.

Survey responses also highlighted ways in which Program grantees have harnessed their funding to inform policy and practice at national and international levels, as outlined in Table 2 [5].

**Table:** Case Study: Research Training and Partnerships in Mental Health

Mental health is a key gap in NCD research and research capacity in many LMICs. FIC Program grantees from the University of Pittsburgh sought to address this gap by leveraging their co-funded National Institute of Mental Health (NIMH) award to develop a program that provides psychiatric research training to investigators in Egypt and India [12]. Through this grant, forty-one trainees were enabled for three months or more to study novel and effective interventions for schizophrenia and other topics including mental functioning, hepatitis C, and mental health-related stigma. Trainees have also partnered with U.S. and LMIC investigators to develop collaborative grant applications for multi-site research studies, further extending the reach of their work. Program funding allowed these researchers to be trained at their home institutions and focus on addressing local mental health concerns. Such a robust project demonstrates the power of collaboration, both among the researchers themselves as well as between NIH Institutes. FIC and NIMH have worked together closely to synergize their approaches to capacity building related to NCDs to help ensure that the best scientific outcomes are developed.

**Table:** Case Study: Clinical, Operational, Health Services, and Prevention Research in India

Over the past decade, FIC-supported grantees from the University of Alabama at Birmingham led a program in India that has trained a cadre of local investigators in clinical, operational, health services, and prevention science research focused on NCDs [13]. In collaboration with Chennai’s Madras Diabetes Research Foundation (MDRF), the program has provided short-term training sessions to an estimated 750 researchers, enabling them to understand the impact of NCDs in India and develop methods to address them. Trainees are taught research methodologies, data management, sample handling systems, basic science and other research skills through a variety intensive training workshops, video conferences, and special courses. The program also created the Indian NCD Network, a formal entity that provides clinical research training on diabetes research and prevention, a major health concern in India. MDRF has evolved into one of the leading diabetes and clinical care institutions in South Asia, and FIC’s training program has helped identify promising young investigators for leadership roles. The program has brought national awareness the problem of NCDs in India, and helped provide the evidence and regional research capacity needed to address it.

**Table 2.** Examples of Policy and Practice Impact

South America
In the Jujuy Province of Argentina, grantees trained primary care health agents on early identification, referral and follow-up of individuals with psychosis, a plan later implemented by the Ministry of Health. The region has since seen a twofold increase in the number of psychologists and psychiatrists in the ministry services. The grantees also participated in an WHO-sponsored meeting to establish a strategic plan and research agenda for national mental health reform in Peru.
East Asia
Program fellows conducted empirical work that informed the drafting of China’s first national mental health law in 2013. A FIC-grant principle investigator was a co-author of the law, and continues to provide information to mental health specialists across the country about the provisions of the law and study the effectiveness of its implementation process. The grantees were recognized in 2012 by the Shanghai Municipal Government’s prestigious Magnolia Silver Award for their dedication to mental health services research, policy advice, and capacity building efforts in the country.
Middle East
FIC-funded U.S. researchers worked alongside Egyptian counterparts to conduct multi-country studies on the highly addictive and abused opioid Tramadol. The evidence collected has informed the WHO and governments of Egypt and the United Arab Emirates about the treatment needs of Tramadol users, resulting in the approval of addiction medications in these countries.

These cases demonstrate the gains that can be made in scientific advances, policy, and practice with research investments that target the global NCD burden. By supporting rigorous and novel research, the Programs have helped advance the science around NCDs in LMICs and provided stakeholders with more data and tools that can be harnessed across sectors to improve NCD-related outcomes.

### Research capacity

As of May 2020, the Programs have provided NCD-specific training for more than 690 LMIC scientists, the majority of whom were long term trainees (407 trained for a duration greater than 6 months), through a combination of opportunities such as fellowships, certificate programs, master’s and doctorate degrees, postdoc positions and workshops for grant writing, lab techniques, implementation science, and research protocol development [14]. The Programs have created a cadre of U.S. and foreign researchers who have successfully leveraged their training to obtain funding to continue NCD research and research training projects in their home countries. Supporting the career development of LMIC scientists so they become independent investigators is critical to the development of a highly skilled and nimble research workforce that can address local health problems including those related to NCDs [15]. By providing a suite of training opportunities, the Programs support a pipeline to scientific independence that can be otherwise difficult for young investigators to obtain in settings where there may be little funding for early career researchers and limited options for mentorship.

In tandem with opportunities to boost individual research capacity, the Programs support the development of LMIC institutional capacity in NCD-related research. Many Program grantees and trainees secured positions in academia and created new research support infrastructure in their home countries such as certificate and degree programs, IRB offices, grant management, and manuscript writing. Alumni reported that their Program funding enabled local institutions to recruit or retain faculty focused on NCD research [5]. Building LMIC institutions to be centers of scientific excellence is an essential component to developing a sustainable research enterprise. Further, the structure of the Programs has encouraged collaboration among U.S. and LMIC researchers and institutions, allowing all partners to capitalize on respective strengths beyond the reach of a single entity. Facilitating collaboration and resource- and information-sharing among institutions allows scientists to make a greater collective impact on complex health problems. This institutional network-building strategy is particularly important in the study of NCDs, a broad field that requires investigators to leverage scientific findings from multiple disciplines.

## Challenges and future needs

Confronting the global NCDs crisis requires a critical mass of scientists who are well versed in regional health problems and understand the cultural, social, economic, and political contexts that influence the effectiveness of interventions to address NCDs. What is considered a ‘critical mass’ of researchers is not consistently defined as it is different for each setting. However, it can be broadly defined as the progress towards a research environment that is a self-sustaining center of excellence in NCD research. FIC is not prescriptive in determining what a country’s needs are, and prospective grantees are required to include an assessment in their application that justifies how their training project will address identified gaps in research capacity. While the Programs have helped build the NCD research capacity in LMICs by training individuals and bolstering research infrastructure, there is still much to be done. Data from a survey of Programs grantees [5] illustrate several important gaps.

### Critical mass researchers and mentors

The increasing diversity of NCDs and co-morbidities adds complexity to research on NCDs as well as the process of building capacity to address NCDs. For example, a country may have built a critical mass of researchers to address cardiovascular disease, but there may remain a lack of experts that can manage the growing diabetes, trauma/injury or hypertension issues in the country. In addition, many surveyed trainees noted that LMICs need more senior researchers who can mentor and assume leadership roles. Moreover, building a sustainable and critical mass of NCD research experts who remain in-country requires viable career paths. Survey data highlight the need for more support for early stage investigators through career development awards targeted specifically toward young researchers.

### Funding information

Data from the Programs suggest there are specific areas of research in the context of NCDs that are neglected by funding opportunities. For example, there is a growing appreciation for the impact of implementation research and many grantees note that understanding how to adapt and scale programs, guidelines, and instruments is critical to addressing NCDs. In addition, there was a call for more support for mental health-related research and training. While recognition of the need for a focus on NCDs has grown, many of the surveyed NCD grantees reported that the funding prospects for conducting NCD training and research remain limited. Grantees stated that because national budgets are too strained to support substantial NCD research, the ability and opportunity for trainees to compete and win in-country NCD-related grants is insufficient.

### Protected research time

Many of the trainees graduating from the Programs return to their institution with the hope of continuing to grow their research careers. However, like many academics, these researchers find themselves with competing interests, forced to split their time among clinical, teaching and research responsibilities. Program grantees noted that it is difficult for faculty to find protected time to conduct research, a problem that is especially true for early career researchers in LMICs.

## Lessons learned

Though adjustments have been made to the Programs since the first iteration launched in 2001, there are still areas for improvement [5]. First, because LMICs face a dual burden of NCDs and infectious diseases, future iterations of the Programs should encourage research and research training at the nexus between the two fields and on co-morbidities. Such a shift would allow investigators’ work to more accurately reflect the increasingly complex public health landscape where diseases intersect and often impact one another. Second, though the Programs have funded a range of NCD-related research, future iterations should prioritize underrepresented topical areas such as metabolic disorders, sight and hearing loss, chronic kidney diseases, and neurological disorders. In addition, the Programs should support research capacity building related to cross-cutting NCD themes such as prevention, implementation science, risk factors, and stigma. Third, as foreign institutions increasingly receive more direct awards, FIC’s annual network meetings should be more easily accessible to LMIC grantees. To date, network meetings have been held on NIH’s campus in Bethesda, Maryland, which is not always a feasible option for those travelling from abroad and can hinder grantees’ and trainees’ opportunities to network with colleagues working in their field. Utilizing virtual platforms and hosting network meetings abroad or in connection with other NCD conferences that grantees are likely to attend are remedies that will help ensure investigators can reap all the benefits of their program, regardless of their location. Finally, and crucially, the Programs should require funded projects to include grant writing as part of their training curricula. Grant writing is a critical skill that should be honed early on to help early-stage investigators become independent researchers. These programmatic adjustments are feasible, would ensure that the Programs are keeping pace with shifts in health research and advancements in technology, and would help improve the quality and returns of capacity building activities.

## Current directions and grant opportunities

Through its investments, FIC seeks to seed and catalyze global NCD research and research training, lay the foundation for sustainable scientific capacity, fill scientific capacity gaps that others can build upon, and seed sustainable collaborations. As NCD research capacity has grown, FIC’s research training model has evolved from a focus on training in the U.S. to training in LMICs led by LMIC scientists. To further support a sustainable NCD research enterprise in LMICs, FIC now also prioritizes direct applications from and awards to LMIC sites. FIC actively encourages collaboration and interaction between LMIC institutions and the development of research training networks. This approach helps more underserved institutions benefit from the investments made and the capacity built in other institutions and expands the trainee pool.

One pathway that allows FIC to amplify the impact of its investments is by synergizing its NCD grant programs with other NIH ICO programs. For example, the National Cancer Institute (NCI) has a complementary program that supports the strengthening of institutional capacity to conduct global cancer research in LMICs **[**16**]**. NCI will accept cancer-focused research training grants from U.S. institutions, and FIC will seek to build on those by accepting direct applications from LMIC institutions. The National Institute of Mental Health has also synergized its programs with FIC over the years, particularly through its collaborative hub programs for international research in mental health **[**17**]**. In collaboration with multiple NIH partners, FIC emphasizes funding the research training of LMIC investigators through its Global Noncommunicable Diseases and Injury Across the Lifespan program **[**18**]** and the career development of young scientists specifically through its Emerging Global Leader Award **[**19**]**. FIC will continue to collaborate with other ICOs and identify programmatic opportunities for synergy across the NIH. We encourage all research training programs to help prepare their trainees for these and other funding opportunities to help build a cadre of NCD scientists in LMICs.

As other ICOs build upon and complement FIC programs, FIC has emphasized more cross-cutting programs and models. The most recent version of the research training funding announcement states that FIC is most interested in and will prioritize research training applications that will 1) cover multiple NCD topics and co-morbid conditions, and/or 2) train in research disciplines applicable across NCD topics, co-morbid conditions and settings, for example (but not limited to) training in epidemiology, demographics, implementation science, genetics/epigenetics, bioinformatics, biomedical engineering, or health technology. Implementation science is an important multidisciplinary field of research that helps catalyze uptake of evidence into policy and practice, and it has become an increasing priority area across the NIH **[**20**]**. The Programs explicitly encourage translational and implementation science training so that investigators are poised to think about how the innovations they develop are evidence-based, appropriate for the context in which they are working, and fit the needs of multiple stakeholders **[**21**]**. Cross-cutting research training programs and associated research projects reflect the increasingly complex nature of chronic health conditions and thus promise to help reduce the global NCD burden. FIC is committed to supporting cross-cutting NCD research and training going forward.

## Conclusion

Building individual and institutional research capacity in LMICs is essential to addressing major health challenges like the rise of NCDs. Local researchers are best poised to address the health needs in their home countries and building this critical mass of scientists is key to addressing regional needs. Yet the fight against NCDs is a global one, and it requires the collaboration of researchers, funders and institutions no matter their location. FIC’s Programs provide a model for sustainable scientific research capacity strengthening and partnership- and network-building that can be applied to effectively address NCDs and other health conditions.

## Paper context

The rising global burden of NCDs necessitates the strengthening of scientific research capacity in LMICs. A set of Programs at the Fogarty International Center of the U.S. National Institutes of Health provides a model for conducting NCD research, building individual and institutional research capacity, and developing sustainable partnerships. This paper highlights findings from the Programs’ review and provides a model that researchers and funders can follow in the fight against NCDs and other health conditions.
